# *Bacillus subtilis* Spores as Vaccine Adjuvants: Further Insights into the Mechanisms of Action

**DOI:** 10.1371/journal.pone.0087454

**Published:** 2014-01-27

**Authors:** Renata Damásio de Souza, Milene Tavares Batista, Wilson Barros Luiz, Rafael Ciro Marques Cavalcante, Jaime Henrique Amorim, Raíza Sales Pereira Bizerra, Eduardo Gimenes Martins, Luís Carlos de Souza Ferreira

**Affiliations:** 1 Vaccine Development Laboratory, Department of Microbiology, Institute of Biomedical Sciences, University of São Paulo, São Paulo, Brazil; 2 Department of Parasitology, Institute of Biomedical Sciences, University of São Paulo, São Paulo, Brazil; Federal University of São Paulo, Brazil

## Abstract

*Bacillus subtilis* spores have received growing attention regarding potential biotechnological applications, including the use as probiotics and in vaccine formulations. *B. subtilis* spores have also been shown to behave as particulate vaccine adjuvants, promoting the increase of antibody responses after co-administration with antigens either admixed or adsorbed on the spore surface. In this study, we further evaluated the immune modulatory properties of *B. subtilis* spores using a recombinant HIV gag p24 protein as a model antigen. The adjuvant effects of *B. subtilis* spores were not affected by the genetic background of the mouse lineage and did not induce significant inflammatory or deleterious effects after parenteral administration. Our results demonstrated that co-administration, but not adsorption to the spore surface, enhanced the immunogenicity of that target antigen after subcutaneous administration to BALB/c and C57BL/6 mice. Spores promoted activation of antigen presenting cells as demonstrated by the upregulation of MHC and CD40 molecules and enhanced secretion of pro-inflammatory cytokines by murine dendritic cells. In addition, *in vivo* studies indicated a direct role of the innate immunity on the immunomodulatory properties of *B. subtilis* spores, as demonstrated by the lack of adjuvant effects on MyD88 and TLR2 knockout mouse strains.

## Introduction

Adjuvants are compounds that enhance the potency, quality or longevity of specific immune responses. The purpose of adding an adjuvant to a vaccine formulation is to enhance the immunogenicity of the co-administered antigens, reduce the number of doses required for protective immunity and improve the efficacy of vaccines in poor responder populations [1], [2]. In general, adjuvants can be classified as delivery systems, immunopotentiators or a combination of both. Delivery systems, such as mineral salts, emulsions and liposomes, efficiently present the antigens to the immune system by aggregating and controlling antigen release. Immunopotentiators, such as cytokines, saponins and Toll-like receptor agonists, increase the immune response to antigens by stimulating the innate immune cells directly [3]–[5]. These adjuvants boost immunity by activating antigen-presenting cells (APCs), which increase the expression of MHC I and/or II molecules, cytokines and the co-stimulatory molecules that are required for T-cell contact and activation. APC maturation determines the magnitude and the type of the T and B cell responses [6].

The identification and development of new adjuvants is a priority since the currently licensed adjuvants for human use do not always induce a potent immune response against different target pathogens, especially in immunologically hyporesponsive populations [4]. Modern adjuvants should induce a strong and balanced immune response with reduced reactogenicity. Furthermore, a good adjuvant should preferentially have a long shelf life and a low production cost [5].

Previous studies have demonstrated that *Bacillus subtilis* spores show a significant adjuvant effect leading to enhanced antibody responses to either co-administered antigens or antigens that are adsorbed on the spore surface [7]–[9]. *B. subtilis* is a gram-positive nonpathogenic and endospore-forming bacterium species that has been used as a tool for different biotechnological applications, such as probiotic formulations for both humans and animals, expression systems for heterologous protein production and vaccine vehicles for the mucosal delivery of antigens [10], [11], [12]. *B. subtilis* can be genetically engineered to express heterologous antigens soon after spore germination or be presented at the spore surface as fusions with spore coat proteins [13], [14]. *B. subtilis* endospores offers unique resistance properties and can survive for long periods of time under a range of stress conditions, such as high temperature, desiccation, absence of nutrients and exposure to chemical solvents. These features facilitate the storage and transport of these endospores [15].

In this study, we evaluated additional details of the immunological mechanisms leading to the adjuvant properties of *B. subtilis* spores. For that purpose, we used the capsid protein gag p24 from HIV-1 (Human Immunodeficiency Virus type 1) as a target antigen. Gag p24 is an immunodominant protein which plays an important role in inducing protective immunity to HIV-1; therefore, it has become an integral component of many vaccine strategies [16], [17]. We investigated whether live or heat-killed *B. subtilis* spores can equally promote specific antibody and cell mediated immune responses to co-administered gag p24 in different mouse models that are immunized via the subcutaneous route. Our findings indicate that the adjuvant effects of *B. subtilis* spores are not affected by the genetic background of the host lineage and require the involvement of the innate immune system for proper stimulation of adaptive immune responses.

## Materials and Methods

### Ethics Statement

All procedures and experiments involving mice were performed with prior approval by the committee on the ethical use of laboratory animals from the Institute of Biomedical Sciences of the University of São Paulo (Protocol #045) and in accordance with the recommendations in the guidelines for the care and use of laboratory animals of the National Committee on the Ethics of Research (CONEP).

### Bacterial strains and spore preparation

The *B. subtilis* WW02 strain (*leuA8 metB5 trpC2 hsd*RM1 *amyE::neo*) was kindly provided by Dr. Wolfgang Schumann from University of Bayreuth, Germany. *B. subtilis* WW02 is a widely used strain, which is a derivative from *B. subtilis* 1012, a reference laboratory strain with a sequenced genome and known physiological features [18], [19]. Spores were prepared from this strain, as previously described [20]. Briefly, sporulation of *B. subtilis* was induced following cultivation in F medium for 72 h at 37°C. The culture was centrifuged, and the pellet was suspended in 50 mM Tris-HCl (pH 7.2) containing 50 µg/mL of lysozyme and incubated for 1 h at 37°C. The spores were then washed sequentially with distilled water, 0.05% SDS and three washes with distilled water. The concentration of the viable spores was determined by plating serial dilutions of the heat-treated samples (60 min at 65°C) on LB plates. Spores were killed by autoclaving at 121°C for at least 30 minutes.

### Purification of recombinant p24

Recombinant p24 with an N-terminal histidine tag was purified by affinity chromatography [unpublished data]. Briefly, *E. coli* BL21 (DE3) cells (Invitrogen) were transformed with the recombinant pET28b/p24 vector and used as the host for protein expression after induction with 0.5 µM isopropyl-β-D-thiogalactopyranoside (IPTG) (Fermentas Life Tecnologies). The cells were harvested 2 h post-induction by centrifugation, and the cell pellet was suspended in buffer A (500 mM NaCl, 100 mM Tris-HCl, 1 mM PMSF, pH 8). The cells were lysed and centrifuged, and the supernatant was clarified with a 0.45 µm mesh membrane filter. Protein purification was carried out in a HisTrap™ HP column (GE Healthcare Bio-Sciences Corp) by immobilized metal affinity chromatography (IMAC) using an AKTA model FPLC equipment (Healthcare Bio-Sciences Corp). The column was washed with buffer A and the bound proteins were eluted using a linear gradient with buffer B (500 mM NaCl, 100 mM Tris-HCl, 1 M Imidazole, pH 8). The peak fractions were dialyzed against phosphate buffered saline (PBS) and concentrated using a membrane filter with a 5 kDa cutoff value (Millipore). The protein concentration was determined using a Bicinchoninic acid (BCA) assay (Pierce) and the purity was monitored by SDS-PAGE. Endotoxin level was equal or lower than 2.0 EU/µg of protein, after its removal by successive washing steps with 1% Triton X-114 [21].

### Adsorption of proteins to spores

Protein adsorption to spores was carried out as previously described [7]. Briefly, 2 µg of the recombinant purified p24 protein was added to a suspension containing 2×10^9^ spores in 0.2 mL of 1X PBS at pH 4, pH 7 or pH 10. The pH was adjusted using HCl or NaOH. After 1 h of incubation at room temperature, the spores were washed 2 times with 1X PBS by centrifugation, and the pellets resuspended in the same buffer. The amount of protein adsorbed to the spore coat, after the washing steps, was quantified by dot-blot assay, as previously described [22].

### Mice and immunization regimens

Pathogen-free BALB/c, C57BL/6, MyD88 and TLR2 deficient mice (C57BL/6 genetic background) at 6–8 weeks of age were used for these studies. The animals were supplied by the Isogenic Mouse Breeding Facilities of the Departments of Parasitology and Immunology, Institute of Biomedical Sciences of the University of São Paulo (USP). Groups of five animals were submitted to an immunization regimen comprising three doses administered via the subcutaneous route on days 0, 14, 21. Mice were inoculated with 700 ng of p24 alone, 700 ng of p24 in combination with 15 µg of Alum, 10 µg of p24 admixed with 1 µg of LT1, 2×10^9^ of live *B. subtilis* spores adsorbed with 700 ng of p24 and 700 ng of p24 mixed with 2×10^9^ of live or heat-killed spores. This mixture was performed using 0.2 mL of 1× PBS at pH 10 and the inoculation of mice was done right after the mixing, in order to avoid any adsorption of the antigen to the spore surface. The control groups received sterile PBS. Sera samples were collected one day before each dose to analyze the antibody production.

### Serum antibody detection

Antigen-specific antibodies in serum were analyzed by ELISA, according to previously described procedures, with minor modifications [23], [24]. Maxisorp plates (Nunc) were coated with purified p24 protein (300 ng/well) diluted in PBS at pH 7.5 and were incubated overnight at 4°C. The wells were washed three times with PBS containing 0.05% Tween 20 (PBS-T) and were blocked with 1% BSA in PBS for 2 h at 37°C. Serum samples were serially diluted in blocking solution and incubated for 1 h at 37°C. Antibody levels were detected using horseradish peroxidase-conjugated rabbit anti-mouse IgG, IgG1, IgG2a or IgG2c (Sigma and Southern Biotech) for 1 h at 37°C. The color was developed with the addition of ortho-phenylenediamine (OPD) substrate, and the absorbance was measured at 492 nm.

### Determination of CD4^+^ and CD8^+^T cell response

Two weeks after the last immunization, the mice were euthanized, and the spleens were removed. The cell suspension was obtained by macerating the spleen and treating the cells with ammonium chloride and potassium (ACK buffer) to lyse the erythrocytes. The spleen cells were stimulated for 16 h, at 37°C in a 5% CO_2_ atmosphere and in the presence of brefeldin-A, with a synthetic peptide (GenScript) corresponding to the CD4 (AMQMLKETINEEAAE) or CD8 (AMQMLKETI) T cell-specific epitope of the HIV gag p24 protein, at a final concentration of 5 µg/mL or 10 µg/mL, respectively. Following incubation, cells were washed twice in PBS supplemented with 2% of fetal bovine serum (FBS) and surface stained with anti-CD8 fluorescein isothiocyanate (FITC) and anti-CD4 Cy (BD BioSciences). Next, the cells were washed, fixed with paraformaldehyde and permeabilized with saponin, and then intracellularly stained for IL-4 or IFN-γ phycoerythrin (PE) (BD BioSciences). FACS analysis was performed on a BD FACSCalibur flow cytometer, and the results were analyzed using FlowJo (Tree Star) software.

### Bone-marrow-derived dendritic cells (BMDC) generation

Murine BMDCs were prepared as formerly described [25], [26] with minor modifications. Briefly, cells were collected from the femoral bone of 6–12 week old C57BL/6 mice, were seeded into non-treated cell culture plates and were cultured at 37°C and 5% CO_2_ for 8–9 days in 10 mL of conditioned medium (RPMI-1640 supplemented with 10% heat-inactivated FBS, 100 U/mL of Penicillin/Streptomycin, 4% non-essential amino acids, 90 µM β-mercaptoethanol, 30 ng/mL murine GM-CSF and 10 ng/mL of IL-4). On day 4, the supernatant was gently removed and replaced with the same volume of pre-warmed conditioned medium. On day 8, BMDCs were collect from the plate and washed once with PBS.

### Effect of *B. subtilis* spores on APCs *in vitro*

BMDCs were treated with PBS, 1 µg of LT1 and live or heat-killed spores at a multiplicity of infection (MOI) of 10000. Twenty-four hours later, the culture supernatants were collected, and the cytokine levels were determined by ELISA using commercially available sets OptEIA (BD Pharmingen). For flow cytometry analysis, cells were collected and stained for 30 min at 4°C with PE-labeled anti-CD11c, APC-labeled anti-CD40 and FITC-labeled anti-MHC I and anti MHC II (BD Pharmingen). After three washing steps, the cells were resuspended in PBS and the samples were analyzed by first gating on CD11c positive cells and then examined for expression of costimulatory molecules. Sixty thousand events were acquired using a FACSCalibur™ (Becton Dickinson) flow cytometer, and the data were analyzed using the FlowJo software (Tree Star).

### Monitoring damages in immunized mice

One day after the third dose, the animals were bled via the retro-orbital plexus for the individual determination of lactate dehydrogenase (LDH) and C-reactive protein (CRP) levels, using analytical kits as recommended by the supplier (Laborclin and Bioclin, Brazil, respectively). Complete blood cell counts were also taken at this time. Whole blood samples were used to evaluate five hematological parameters: white blood cells (WBC), monocytes (MON), lymphocytes (LYM), neutrophils (NEU) and eosinophils (EOS). WBC counts were carried out using a Neubauer chamber and MON, LYM, NEU and EOS counts were performed using a phase contrast microscope (Eclipse E200 model, Nikon) [27].

### Statistical analyses

Statistical significance (*p*<0.05) was determined using two-way ANOVA followed by the Bonferroni posttest. The mean fluorescence intensity (MFI) data were analyzed by Student's *t* test. Analyses were conducted using GRAPHPAD PRISM 5 software and were expressed as the means ± standard error of mean (s.e.m.).

## Results

### The adjuvant effects of co-administered *B. subtilis* spores are independent of host genetic background

**Figure 1:**
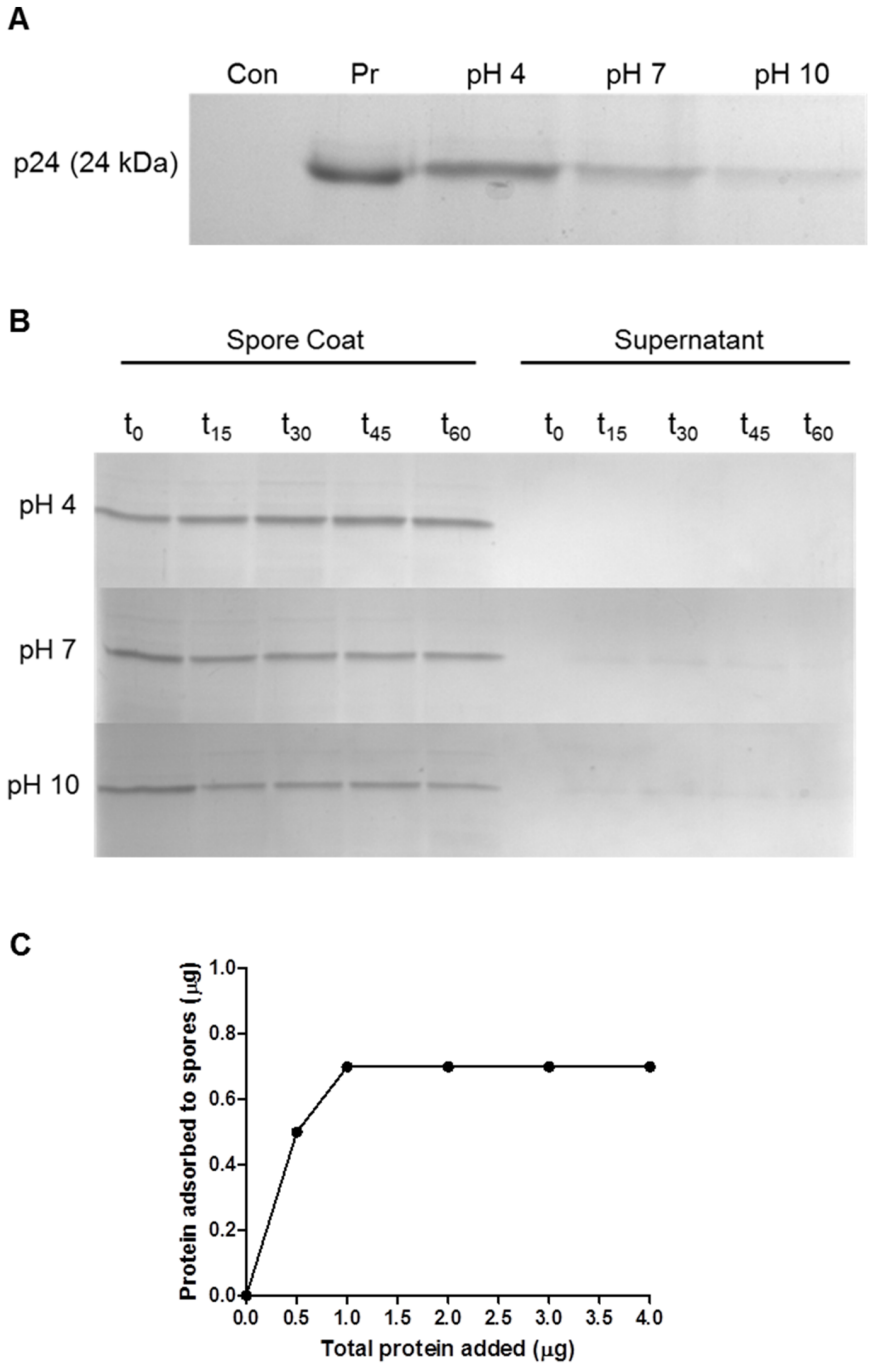
Adsorption of the p24 protein to spores of the *B. subtilis* WW02 strain. (A) Evaluation of protein adsorption to spores at different pHs. Spores (2×10^9^) were suspended in PBS buffer at pH 4, pH 7 or pH 10, mixed with purified p24 protein (2 µg) and kept for 1 h at room temperature. The samples were centrifuged, washed twice with PBS and the spore coat proteins were extracted and submitted to electrophoresis in SDS-PAGE at 15%. The control lane (Con) represents spore samples without incubation with the protein and the control lane (Pr) shows 2 µg of purified p24. Image is representative of four independent experiments. (B) Detection of protein released from spores after adsorption. Spores adsorbed with p24 (PBS at pH 4) were resuspended in PBS at pH 4, pH 7 or pH 10 and incubated for different times. At indicated time points (15, 30, 45, and 60 min) samples were centrifuged and the supernatants and pellet (spores) were loaded onto 15% SDS-PAGE gel. (C) Determination of the saturation curve of p24 protein adsorption to *B. subtilis* spores. Increasing amounts of protein (0.5 to 4 µg) were added to *B. subtilis* spores (2×10^9^) following the procedure described before. The amount of protein adsorbed to the spores coat was quantified by dot-blot assay.

**Figure 2:**
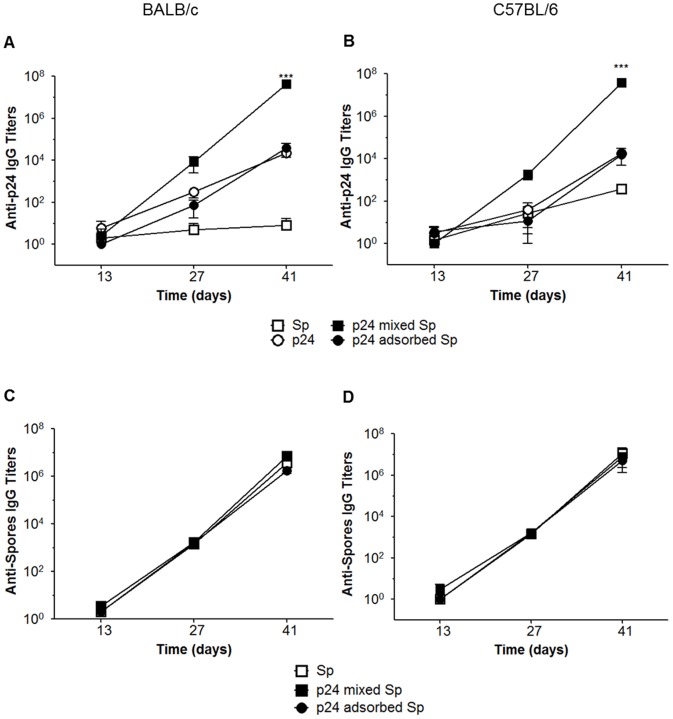
Evaluation of antibody responses following subcutaneous immunization with p24 protein adsorbed or mixed with spores. Mouse groups (n = 5) were immunized (three doses on days 0, 14 and 28) with 2×10^9^ spores (□), 700 ng of p24 only (○), 700 ng of p24 in combination with 2×10^9^ spores (▪) or 700 ng of p24 adsorbed at 2×10^9^ spores (•). Levels of anti-p24 (A and B) and anti-spores (C and D) specific IgG antibodies were detected in serum samples at 13, 27 and 41 days after immunization in BALB/c and C57 BL/6 mice. The results are presented as the mean ± s.e.m. of three independent experiments, and the values obtained from PBS immunized mice were subtracted from each group. *** *p*<0.001 when compared with mice immunized with p24 only.

*B. subtilis* spores have been used as particulate adjuvants for enhancing immune responses based in two main approaches: co-administration with soluble antigens or with proteins adsorbed onto the spore surface [7], [8]. To verify which strategy would confer stronger adjuvant effects, we used the recombinant HIV-1 gag p24 protein, which has been used in several anti-HIV vaccine strategies as a model antigen [23], [24], [28]. BALB/c and C57BL/6 mice were immunized with 700 ng of p24 protein alone, co-administered with or adsorbed onto 2×10^9^ *B. subtilis* spores. Although we had successfully adsorbed the protein onto the spore surface and obtained the maximum adsorption value (700 ng of p24) at pH 4 (Fig. 1), the immunization experiments demonstrated no significant adjuvant effect either in BALB/c or C57BL/6 mice (Fig. 2A, 2B). However, co-administration of the protein with spores promoted a significant increase in the antibody titers against p24 in both mice strains, demonstrating that the adjuvant effects are not affected by the genetic background of the host. Additionally, we observed that antibody responses against the spores were generated in all experimental groups with no difference between the two strategies (Fig. 2C, 2D). This leads us to believe that the protein adsorption strategy does not favor the adjuvanticity of spores regarding the humoral immune responses.

### Live and heat-killed spores can be used as parenteral adjuvants

**Figure 3:**
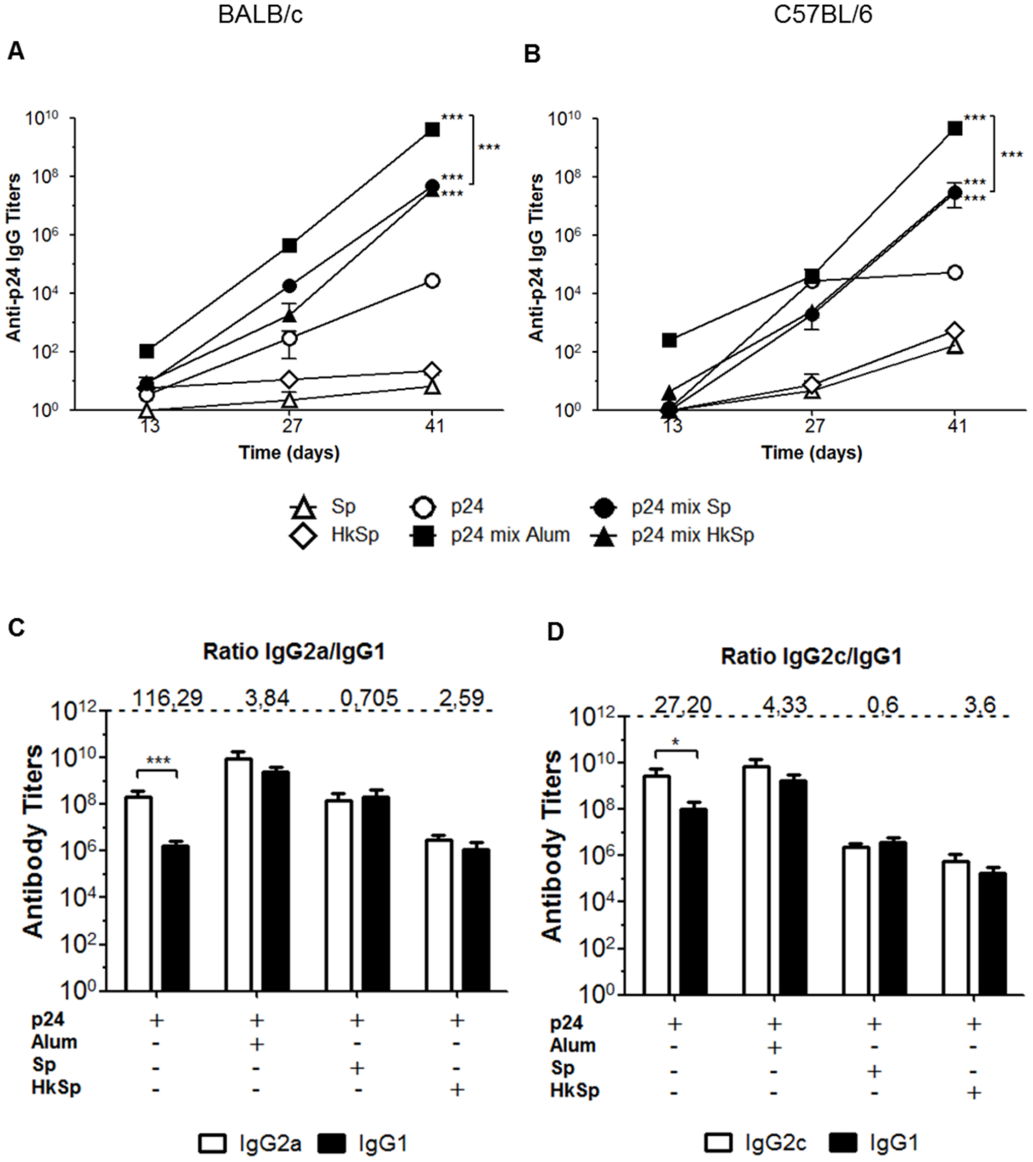
Humoral responses after subcutaneous immunization with p24 protein mixed with live or heat-killed spores. Detection of anti-p24 specific IgG antibodies in serum samples of immunized BALB/c (A) and C57 BL/6 mice (B). Mouse groups (n = 5) were immunized (three doses on days 0, 14 and 28) with viable spores (**▵**), heat-killed spores (HkSp) (**◊**), p24 protein (○), p24 protein in combination with Alum (▪), p24 protein in combination with spores (•) or p24 protein in combination with heat-killed spores (▴). *** *p*<0.001 when compared with group immunized with p24 only or with each other. Specific IgG subclasses elicited in BALB/c (C) and C57BL/6 (D) mice after complete immunization regimen. The IgG2a/IgG1 or IgG2c/IgG1 ratios are indicated at the top of the figure. * *p*<0.05 or *** *p*<0.001. All the results are presented as the mean ± s.e.m. of three independent experiments, and the values obtained from PBS immunized mice were subtracted from each group.

**Figure 4:**
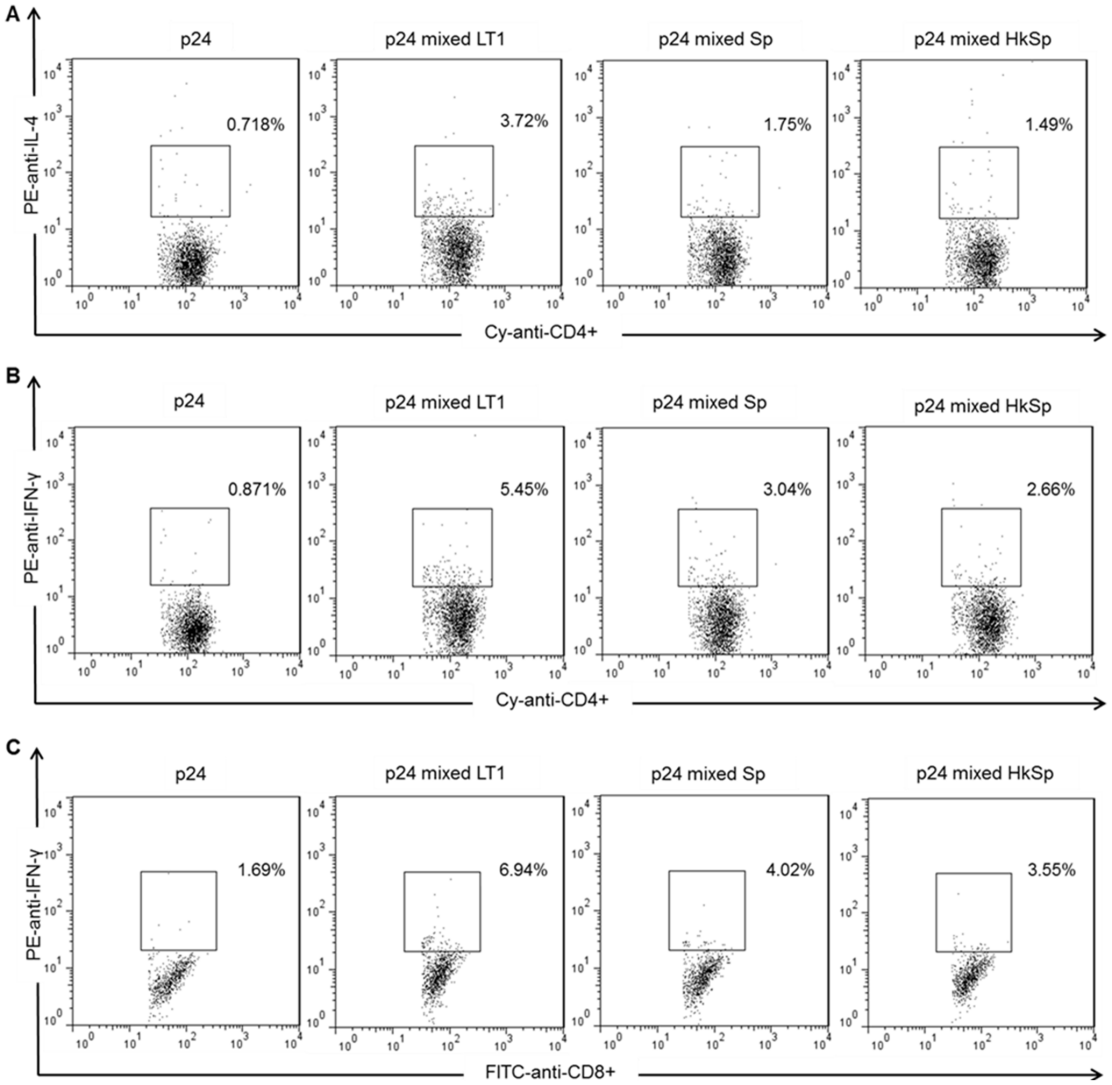
Intracellular cell staining and flow cytometry analysis of p24-specific CD4^+^ and CD8^+^ T cells. BALB/c mice were immunized (three doses on days 0, 14 and 28) with p24 protein (700 ng), p24 (10 µg) admixed with LT1 (1 µg), p24 (700 ng) admixed with spores (2×10^9^) or p24 (700 ng) admixed with heat-killed spores (2×10^9^). Spleen cells were collected two weeks after the last dose, and the detection of IL-4 producing p24-specific CD4^+^ T cells (A) and IFN-γ producing p24-specific CD4^+^ (B) and CD8^+^ T cells (C) was carried out after stimulation with MHC class I or II restricted p24 peptide and cell surface staining for CD8 (FITC), CD4 (Cy) and intracellular staining for IL-4 or IFN-γ (PE). The percentage of each population is indicated in the upper right corners and the frequencies in all groups in the absence of stimulus were below 0.70% (data not shown). Measurements were performed in duplicate for each individual sample, and the data are representative of two independent experiments.

We tested whether the co-administration of heat-killed spores could enhance antigen-specific antibody responses. The results indicate that both live and heat-killed spores induce similar serum anti-p24 IgG levels in both mice strains (Fig. 3A, 3B). A similar behavior was also observed after determining the antigen-specific IgG subclass responses. After the third dose, administration of p24 alone induced an elevated IgG2a/IgG1 and IgG2c/IgG1 ratios, indicating a more Th1-polarized response. On the other hand, mice immunized with p24 co-administered with spores reduced to IgG2a/IgG1 ratio, ranging from 0.7 to 2.59, and IgG1/IgG2c ratio, ranging from 0.6 to 3.6 (Fig. 3C, 3D). The reduction of IgG2a/IgG1 and IgG2c/IgG1 ratios was also observed in mice immunized with p24 in combination with Alum, an adjuvant associated with the induction of Th2 responses [29]. In order to investigate if the balanced distribution of IgG1 and IgG2 isotypes in mice immunized with p24 co-administered with spores was indicative of generation of both Th1 and Th2 responses, the activation of CD4^+^ and CD8^+^ T cell responses were measured in the spleen cells harvested two weeks after the last immunization dose. Co-administration of live or heat-killed spores induced a stronger activation of p24-specific CD4^+^ T cells, which were capable of producing IL-4 or IFN-γ, than the mice immunized with p24 only (Fig. 4A, 4B). Mice immunized with 700 ng of p24 co-administered with spores also showed higher p24 specific CD8^+^ T cell responses than the mice immunized with p24 only (Fig. 4C). Considering that mice immunized with p24 in combination with Alum did not elicit a T cell response higher than mice immunized with p24 only (data not shown), we used, as a control, 10 µg of p24 admixed with 1 µg of heat-labile toxin type 1 (LT1) produced by enterotoxigenic *Escherichia coli* previously characterized by the significant humoral and cellular adjuvant effects [30].

### Safety evaluation of *B. subtilis* as parenteral adjuvant

To develop a new vaccine adjuvant for human or veterinary use, it is important to evaluate the safety of the vaccine in preclinical studies. To evaluate the safety of *B. subtilis* spores as vaccine adjuvant, we examined the hematological profile (white blood, monocytes, lymphocytes, neutrophils and eosinophils) of mice immunized with three doses of live or heat-killed spores. As indicated in Table 1, no evidence of hematologic disturbances was observed after the subcutaneous administration of the spores. We also evaluated the physical changes (hair loss and local inflammation), the histologic alterations of the inguinal lymph nodes and the non-specific biochemical markers of inflammatory reactions. No differences were found between the control group (immunized subcutaneously with PBS) and the group immunized with spores (data not shown). These data indicate that *B. subtilis* spores, in the complete immunization regimen via the subcutaneous route, are safe for use as a promising adjuvant.

**Table 1:** Hematological analyses of mice immunized with *B. subtilis* spores^a^.

Hematological	BALB/c			C57BL/6		
parameters^b^	PBS	Sp	HkSp	PBS	Sp	HkSp
**WBC**	55±31	58±13	40±12	61±11	50±17	58±11
**MON**	3.8±3	1.3±1.1	1.5±1.4	1.8±0.9	1.6±1.3	0.8±0.4
**LYM**	54±17	41±9	28±6.3	46±9.3	35±9.1	47±10
**NEU**	10±2.2	15±4.4	7.1±1.8	7.3±3.1	13±8.6	9.7±1.1
**EOS**	0.9±0.6	0.6±0.1	3.1±3.2	4.6±1.7	0.2±0.3	1.1±0.7

a Five mice per immunization group were bled 1 day following the final dose.

b Blood samples were processed to determine white blood cells (WBC), monocytes (MON), lymphocytes (LYM), neutrophils (NEU) and eosinophils (EOS). All the counts are given in 10^2^ cells/µl.

Data are expressed as the mean ± SD of individual measurements. Differences among groups were not observed after ANOVA and Bonferroni's Multiple Comparison test. The results in this table represent one of two independent experiments.

Sp: *B. subtilis* WW02 spores; HkSp: Heat-killed *B. subtilis* WW02 spores.

### Maturation of dendritic cells by *B. subtilis* WW02 spores

**Figure 5:**
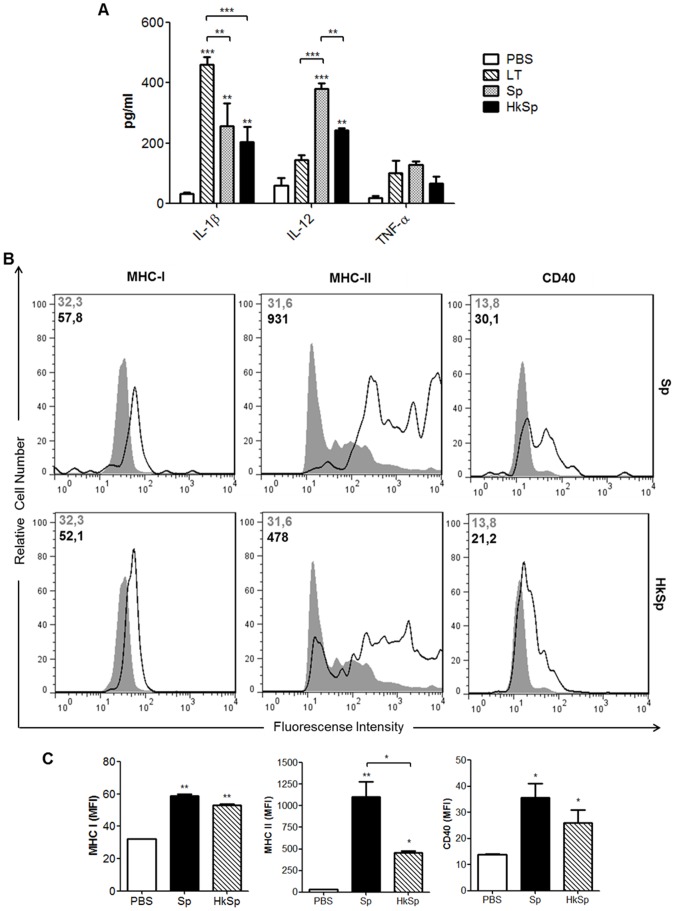
Maturation of dendritic cells after stimulation with *B. subtilis* spores. (A) Induction of pro-inflammatory cytokines after stimulation with spores. BMDCs from C57BL/6 mice were treated with PBS (control group), LT1 and live or heat-killed spores at a MOI of 10.000. Supernatants were collected after 24 h, and the IL-1β, IL12p70 and TNF-α concentrations were quantified by ELISA. (B) Expression of surface molecules by CD11c^+^ cells following exposure to spores. BMDCs were treated as described above and the surface expression levels of CD40, MHC-I and MHC-II were measured by flow cytometry. Gray-filled histograms represent samples incubated with PBS and open histograms represent samples incubated with spores. The MFI for both conditions is shown in the upper left in each histogram. (C) Graphical comparison of the expression of CD40, MHC-I and MHC-II. Data represent mean values ± s.e.m. of two independent experiments. *p<0.05, **p<0.01 or *** *p*<0.001 when compared with control group or with each other. Sp: *B. subtilis* WW02 spores; HkSp: Heat-killed *B. subtilis* WW02 spores.

Surface expression of co-stimulatory molecules and cytokine secretion are some of the features observed during activation of antigen presenting cells, such as dendritic cell (DC), a key step in the generation of an adaptive immune response. A previous report demonstrated that dead *B. subtilis* spores can enhance the expression of MHC-II molecules and the secretion of IL-12 by dendritic cells [9]. In this study, BMDCs were treated with live or heat-killed spores of the *B. subtilis* WW02 strain to determine if spores would induce the maturation of dendritic cells by enhancing the expression of the surface molecules and the secretion of cytokines. Levels of IL-12, TNF-α and IL-1β detected in supernatants collected from DC cultured *in vitro* were significantly increased after treatment with spores, and the live spores induced a higher secretion of IL-12 than the heat-killed spores (Fig. 5A). FACS analysis of dendritic cells treated with spores revealed increased surface expression of MHC I, MHC II and CD40 molecules, although live spores were more effective in inducing MHC II surface expression (Fig. 5B, 5C). These results indicate that spores can induce DC activation and production of pro-inflammatory cytokines.

### The innate immunity and the adjuvant effects of *B. subtilis* spores

**Figure 6:**
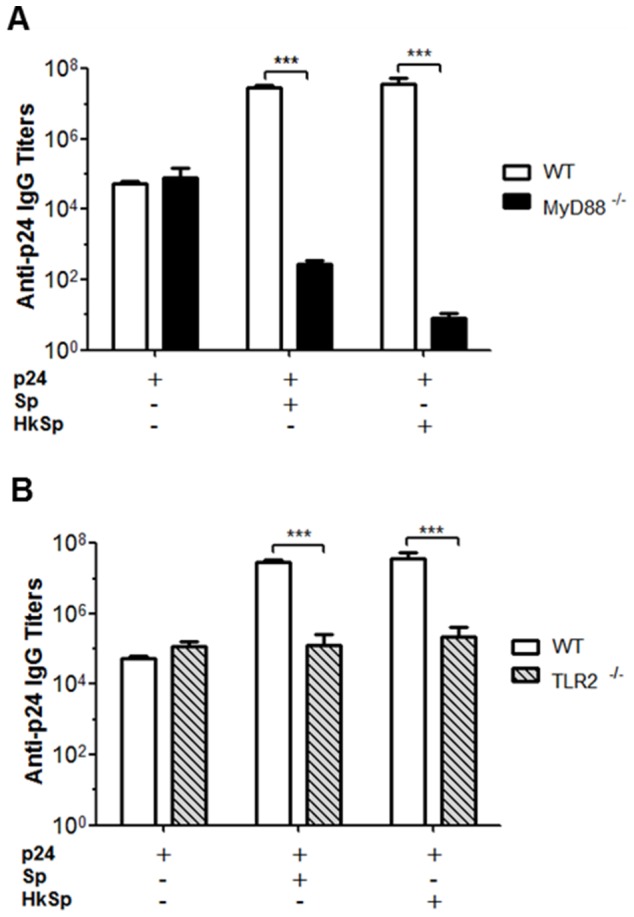
The adjuvant effects of *B. subtilis* spores require stimulation of TLR2. MyD88^−/−^ (A) and TLR2^−/−^ (B) C57 BL/6 mice (n = 5) were immunized (three doses on days 0, 14 and 28) with p24 protein only (700 ng) or p24 (700 ng) in combination with live or heat-killed spores (2×10^9^). Levels of anti-p24 specific IgG antibodies were detected in serum samples 13 days after the third dose. The results represent the means ± s.e.m. of two experiments, and the values obtained from PBS immunized mice were subtracted from each group. *** *p*<0.001.

DC can express several pattern recognition receptors including the toll-like receptors, which can recognize different molecular patterns that are common in microorganisms. Signals generated from various toll-like receptors determine the nature of immune responses, and the ligands of toll-like receptors are potent stimulators of the adaptive immune system [31]. We evaluated whether live and heat-killed *B. subtilis* spores interact with toll-like receptors and can synergistically potentiate vaccine-induced responses. First, we analyzed if the signaling pathway that is dependent on the adapter protein MyD88 is related to the adjuvant effect of the spores during the humoral response to p24. Wild type and MyD88 knockout C57BL/6 mice received 700 ng of p24 protein alone or co-administered with 2×10^9^ of live or heat-killed spores. In mice immunized with protein p24 only, the humoral immune response was not affected by MyD88 inactivation. However, MyD88 knockout mice did not show enhancement of the p24-specific antibody response after co-administration with either live or heat-killed *B. subtilis* spores (Fig. 6A). Because the MyD88 signaling pathway is involved in the adjuvant effect of spores, we decided to determine which TLR plays a role in this process. Considering that *B. subtilis* vegetative cells and spores can stimulate the *in vitro* expression of TLR2 by dendritic cells [32], we immunized TLR2 knockout C57BL/6 mice with different vaccine formulations. TLR2 knockout mice were not able to elicit a strong systemic antibody response to the antigen with regard to the wild type mouse strain when the protein was co-administered with *B. subtilis* spores (Fig. 6B). These data indicate that the spores interact with DCs via TLR2 and promote maturation of these cells, leading to enhanced adaptive immune responses.

## Discussion

The limited immunogenicity of most purified vaccine antigens has raised interest for new adjuvants with enhanced efficacy and safety. Previous studies have shown that *B. subtilis* spores can enhance the immune response against protein antigens and represent a potential candidate for a novel particulate adjuvant. Nonetheless, a better understanding of the adjuvant activity of *B. subtilis* spores is crucial to design more effective vaccine formulations. The results described in this study clarify the mechanisms underlying the induction of the humoral and cellular immune responses to live and heat-killed spores that are subcutaneously administered to mice.

Initially, we compared the effectiveness of two previously reported approaches in which spores were used as vaccine adjuvants [7], [8]. The first approach consisted of the adsorption of proteins onto the spore surface, and the second approach consisted of the co-administration of the spores with the antigen. Our results demonstrated that, in the two tested mouse strains, co-administration of spores result in a better adjuvant effect on the induced antigen-specific antibody response when compared with the results generated in mice immunized with spores in which the antigen was adsorbed on the spore surface. Because the anti-spore antibody responses were similar in both immunization approaches, the lower adjuvanticity recorded with antigen-adsorbed spores could not be attributed to any impairment of the immunization procedure. Although previous studies have shown that the adsorption of proteins onto the spore coat is an effective strategy to increase the antibody response against soluble antigens following mucosal administration [7], we could not confirm this effect using a parenteral administration route.

The genetic background of the mouse strain may affect the Th profile of the induced immune responses in animals submitted to an immunization regimen. C57BL/6 mice characteristically show a Th1 immune response pattern with high production of INF-γ by T cells, whereas BALB/c mice develop a Th2 biased immune response with high production of IL-4 [33]. Previous studies have shown that *B. subtilis* spores can induce a balanced Th1 and Th2 response pattern [8]. In this study, we further confirmed the same immune response pattern induced in both C57BL/6 and BALB/c mouse strains. In addition, specific antibody production and activation of antigen specific IL-4 producing CD4^+^ T cells and IFN-γ producing CD4^+^ T and CD8^+^ T cells were detected after immunization with *B. subtilis* spores. Collectively, these results strongly suggest that spores can elicit both humoral and cellular immune responses which are independent of the host genetic background when administered via parenteral route.

Alum was more efficient to increase the humoral responses against p24 than spores, but failed to increase cellular immune responses to the same antigen. LT1, on the other hand, performed better than spores to induce CD4^+^ and CD8^+^ T cell responses, but only at experimental conditions in which the antigen was used at a significantly higher concentration. Live and heat-killed spores were equally effective in inducing an increase in the humoral and cellular response against p24, using a lower dose of antigen. Besides the fact that heat-killed spores are as efficient as live spores is a feature that would further facilitate the handling and storage of this adjuvant.

Effective stimulation of T cells requires appropriate signaling provided by matured dendritic cells, such as increased expression of costimulatory molecules and secretion of cytokines, and the presentation of antigenic peptides bound to MHC molecules [6]. Our *in vitro* studies showed that live and heat-killed spores can induce the maturation of dendritic cells, increasing the expression of pro-inflammatory cytokines and MHC-I, MHC-II and CD40 molecules, although live spores were more effective than heat-killed spores in inducing MHC-II surface expression and IL-12 secretion. Thus, such increased activation of dendritic cells by live *B. subtilis* spores may be attributed to spore germination, either in the culture medium or inside the cells following phagocytosis, as previously demonstrated to occur [32].

DCs can respond to foreign organisms, initiating an intracellular signaling pathway mediated by the TLR receptor that leads to the production of pro-inflammatory cytokines and costimulatory molecules that link innate and adaptive immunity. MyD88 knockout mice failed to enhance the p24 specific antibody response in the presence of the spores, indicating that the signaling pathway operates through the MyD88 signaling pathway to exert the adjuvant effects in mice. Although the MyD88-dependent pathway is utilized by most of TLRs, our results provide evidence that adjuvanticity of *B. subtilis* spores results from recognition by Toll-like receptor 2 because the adjuvant effect disappeared in immunized TLR2 knockout mice.

Safety issues represent a major challenge in the search of new adjuvants for animals and humans. Our results indicate that *B. subtilis* spores can be administered via parenteral routes without causing measurable side effects and, therefore, can be regarded as safe for parenteral inoculation. No alterations in biochemical and hematological parameters were detected following immunization with the spores, indicating that the subcutaneous administration of a high amount of spores does not pose major safety concern issue.

In summary, our study significantly advances the current understanding of the mechanisms involved with the adjuvanticity of *B. subtilis* spores. We demonstrated that parenteral co-administration of live and killed spores can safely induce an increase of the specific antibody and T cell response to HIV gag p24 antigen in both BALB/c and C57BL/6 murine models. In addition, the spores stimulated DCs to express pro-inflammatory cytokines and costimulatory molecules via TLR2 and MyD88 signaling pathway.
